# Cautions as to Health Resorts

**Published:** 1898-06-18

**Authors:** 


### CAUTIONS AS TO HEALTH RESORTS.

At a conversazione given on the 8th inst. by the British Balneological and Climatological Society, Dr. G. Vivian Poore delivered a characteristic and witty address on "Circumstances Influencing the Effectiveness of Climatic Treatment." He pointed out the unwisdom of sending a patient to a spot with which the medical man had no personal acquaintance, seeing that two places might be within a mile or so of each other, and yet have totally different climates. Another frequent mistake was to select an expensive health resort for a patient of limited means, thus neutralising the climatic advantages by enforced inferior housing and feeding. The suitability of the place must also be studied; he instanced the case of a country squire immured in Homburg, without understanding a word of French or German, and at the same time compelled to consume food and follow habits which were thoroughly distasteful to him. Dr. Poore was particularly trenchant in his criticism of hotels, both English and foreign. The modern system of piling storey upon storey was, from the sanitary point of view, essentially bad; a hotel should be built like an infectious hospital or an Oxford college, and the common rooms should be in a different part to the bedrooms. The food, too, was frequently abominable—neither more nor less than "disguised garbage"—and when to these disadvantages were added the seasonal overcrowding and the noisy attractions offered to cheap trippers, it was hard to tell whence the benefit to health was to be derived. But, of all the warnings he gave, none was more explicit or valuable than that as to the selection of medical attendants upon patients sent to Continental health resorts. At home, one's choice is to a certain extent safeguarded by the Medical Register and Directory, but health resorts abroad are the abodes of hordes of quacks, into whose clutches only too many English patients fall. The obvious remedy is to send only to

British practitioners, but, unfortunately, most Continental governments restrict their power of carrying on their profession. This lack of reciprocity will, however, eventually cut both ways, for it must of necessity lead to the opening up of the unrivalled health resorts of the British colonies. Egypt and South Africa have already begun to reap the benefit, and Australia, New Zealand, and Canada will no doubt soon do so. One of the great works of the Society will certainly be to open up the climatological capacities of the British Empire.

